# Capping Enzyme mRNA-cap/RNGTT Regulates Hedgehog Pathway Activity by Antagonizing Protein Kinase A

**DOI:** 10.1038/s41598-017-03165-2

**Published:** 2017-06-06

**Authors:** Ping Chen, Zizhang Zhou, Xia Yao, Shu Pang, Meijing Liu, Weirong Jiang, Jin Jiang, Qing Zhang

**Affiliations:** 1 State Key Laboratory of Pharmaceutical Biotechnology and MOE Key Laboratory of Model Animals for Disease Study, Model Animal Research Center of Nanjing University, Nanjing, 210061 China; 2 Department of Molecular Biology, University of Texas Southwestern Medical Center at Dallas, Dallas, TX 75390 USA; 3 Department of Pharmacology, University of Texas Southwestern Medical Center at Dallas, Dallas, TX 75390 USA

## Abstract

Hedgehog (Hh) signaling plays a pivotal role in animal development and its deregulation in humans causes birth defects and several types of cancer. Protein Kinase A (PKA) modulates Hh signaling activity through phosphorylating the transcription factor Cubitus interruptus (Ci) and G protein coupled receptor (GPCR) family protein Smoothened (Smo) in *Drosophila*, but how PKA activity is regulated remains elusive. Here, we identify a novel regulator of the Hh pathway, the capping-enzyme mRNA-cap, which positively regulates Hh signaling activity through modulating PKA activity. We provide genetic and biochemical evidence that mRNA-cap inhibits PKA kinase activity to promote Hh signaling. Interestingly, regulation of Hh signaling by mRNA-cap depends on its cytoplasmic capping-enzyme activity. In addition, we show that the mammalian homolog of mRNA-cap, RNGTT, can replace mRNA-cap to play the same function in the *Drosophila* Hh pathway and that knockdown of *Rngtt* in cultured mammalian cells compromised Shh pathway activity, suggesting that RNGTT is functionally conserved. Our study makes an unexpected link between the mRNA capping machinery and the Hh signaling pathway, unveils a new facet of Hh signaling regulation, and reveals a potential drug target for modulating Hh signaling activity.

## Introduction

The Hedgehog (Hh) signaling pathway is essential for embryonic development and adult tissue homeostasis in species ranging from insects to mammals^1–3^. Dysregulation of Hh signaling has been implicated in a large number of human disorders, including birth defects and a wide range of cancers^4–7^.

Hh signal transduction is best studied in *Drosophila*. In the absence of Hh, the 12-transmembrane protein Patched (Ptc) inhibits the function of the 7-transmembrane GPCR family protein Smoothened (Smo), which transduces the Hh signal across the plasma membrane to intracellular signaling components^8–14^. When Hh binds Ptc, the inhibition on Smo is relieved. Activated Smo recruits the kinesin-like protein Costal2 (Cos2) and the Ser/Thr kinase Fused (Fu) to trigger the activation of latent transcription factor Cubitus interruptus (Ci)^15–22^, which enters the nucleus to turn on the expression of Hh target genes including *decapentaplegic (dpp)*, *patched (ptc)* and *engrailed (en)* ^1, 23, 24^. The expression of different responsive genes is dependent on the Hh dosage. Low levels of Hh are able to induce the expression of *dpp*, intermediate to high levels of Hh are required to activate *ptc*, and peak levels of Hh are required to activate *en* ^25^.

In *Drosophila* wing discs, when Hh levels are very low or absent in anterior (A) compartment cells far away from the anteroposterior (A/P) compartment boundary, the full-length Ci (Ci^F^) undergoes sequential phosphorylation by PKA, GSK3 and casein kinase 1 (CK1) at multiple Ser/Thr residues, which targets Ci^F^ for SCF^Slimb^-mediated proteolytic processing to generate a truncated repressor form (Ci^R^)^26–31^. Ci^R^ enters the nucleus to block the expression of Hh target genes, including *dpp* ^23, 24, 28, 32–35^. Among all the Ci kinases, PKA plays a primary role because its phosphorylation of Ci provides priming sites for GSK3- and CK1-mediated phosphorylation^23, 27, 33, 36^.

In A-cells near the A/P boundary where Hh is present, Hh induces Smo activation by promoting phosphorylation of its C-terminal tail by PKA and CK1, which increases Smo cell-surface expression and converts Smo from a closed, inactive conformation to an open and active one^14, 37–39^. Activated Smo inhibits Ci processing and converts Ci^F^ into an active form (Ci^A^) that activates the Hh target genes including *ptc* and *en* ^40, 41^. Hence, PKA plays a dual role in the Hh pathway by activating Smo but inhibiting Ci depending on the availability of the Hh ligand. Despite its dual role in Hh signaling, loss of PKA results in Hh pathway activation instead of inhibition due to the depression of Ci: loss of Ci^R^ and accumulation of Ci^F^, some of which is converted into Ci^A^ ^24, 42^.

*Drosophila mRNA-cap* is a capping enzyme that harbors a RNA 5′-triphosphatase and mRNA 3′-guanylyltransferase domains and catalyzes the attachment of the 5′ cap to messenger RNA molecules in the nucleus during the first stage of gene expression^43–48^. Cap removal is considered to be a prerequisite step in the mRNA degradation pathway^49^, but recent findings indicate that certain portion of translational inactive mRNAs might be stored in an uncapped state and subsequently returned to an active state upon cytoplasmic re-capping^50^. In this case, capping enzyme cooperates with a novel kinase to generate capped ends from cleaved RNAs in cytoplasm, which is named cytoplasmic capping. Except for its capping function, other functions of mRNA-cap are elusive. In this study, we find unexpectedly that *mRNA-cap* regulates Hh signaling activity depending on its capping-enzyme activity in the cytoplasm. Furthermore, we find that it regulates Hh signaling through antagonizing PKA.

## Results

### Downregulation of mRNA-cap disrupts wing development

**Figure 1:**
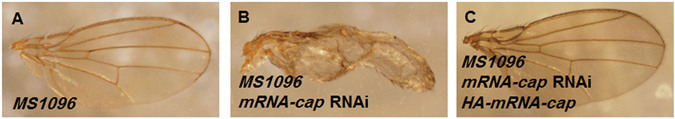
Knockdown of *mRNA-cap* impairs wing development. (**A**–**C**) Adults wing phenotypes from control (**A**), *mRNA-cap* knockdown without (**B**) or with (**C**) *HA-mRNA-cap* overexpression by the *MS1096* Gal4 driver. All images are representative of more than three independent experiments.

Given that Hh pathway plays an important role in wing development and its deregulation causes wing development defect, we sought to identify novel regulators of Hh signaling via analyzing wing phenotypes caused by either loss or gain of function of such regulators. To do that, we performed *in vivo* RNAi screen by silencing ~7000 genes that are conserved between *Drosophila* and mammals using a wing specific Gal4 driver *MS1096*-Gal4 and determined which genes modulated wing development. We picked up the candidate genes whose knockdown caused abnormal wing phenotypes, and further investigated whether they modulated Hh signaling activity by examining the pathway target gene *ptc*-lacZ via immunostaining. In our screen, we found that knockdown of *mRNA-cap* through its RNAi line (VDRC 108809) disrupted wing development, causing deformed wing phenotype (Fig. 1A,B). To verify the efficiency of this RNAi line and rule out any off-target effect, we made *UAS-HA-mRNA-cap* transgenic flies. Through co-expressing the transgene and the RNAi line, we found that mRNA-cap transgenic expression could rescue the RNAi-induced abnormal wing phenotype (Fig. 1C), suggesting that the aberrant wing phenotype is indeed caused by loss of *mRNA-cap*. We also employed another *mRNA-cap* RNAi line (Bloomington 32847), which is a relatively weaker line compared with 108809, and observed a similar wing defect (Supplementary Fig. 1A), and this defect was also rescued by overexpressing the *mRNA-cap* transgene (Supplementary Fig. 1B). Taken together, these results indicate that mRNA-cap is essential for normal wing formation in *Drosophila*.

### Knockdown of mRNA-cap represses Hh signaling activity

**Figure 2:**
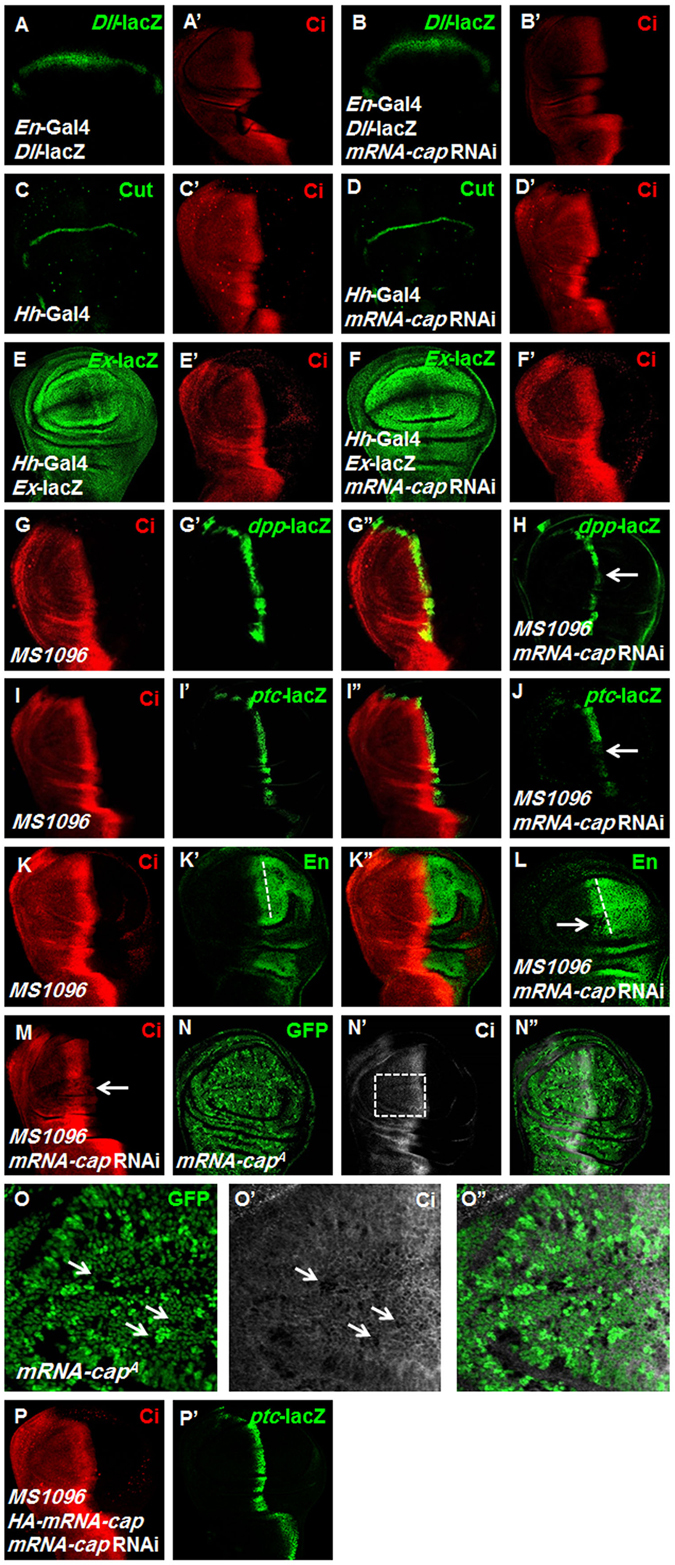
Knockdown of *mRNA-cap* decreases Ci protein level and downregulates Hh target genes expression. All wing imaginal discs shown in this study were oriented with anterior compartments on the left and ventral regions on the top. (**A**–**B’**) Knockdown of *mRNA-cap* with *En-Gal4* (**B**,**B’**), which drives *UAS* target gene expression in the P-compartment, did not decrease the *Dll*-lacZ level compared with control (**A**,**A’**). (**C**–**D’**) The expression of Cut was not decreased compared with control (**C**,**C’**) upon knockdown of *mRNA-cap* in the P-compartment with *Hh-Gal4* (**D**,**D’**). (**E**–**F’**) Knockdown of *mRNA-cap* with *Hh-Gal4* (**F**,**F’**) did not change the expression of *Ex-*lacZ compared with control (**E**,**E’**). (**G**–**M**) Knockdown of *mRNA-cap* with *MS1096* attenuated the expression of *dpp*-lacZ (compare **G’** with **H**), *ptc-*lacZ (compare **I’** with **J**), En (compare **K’** with **L**) and Ci (compare **M** with **G**). Arrows indicate the decrease of *dpp-*lacZ, *ptc*-lacZ, En and Ci. (**N**–**O**”) *mRNA-cap* ^*A*^ clones with low (**N–N**”) and high (**O**–**O**”) magnifications of wing discs. The wing discs were immunostained to show the expression of GFP (green) and Ci (white). *mRNA-cap* ^*A*^ clones were recognized by the lack of GFP (O, arrows). Images in (**O**–**O**”) are enlarged views of the region marked by dashed lines in N’. Of note, *mRNA-cap* mutant cells exhibited decreased Ci level (**O’**, arrows). (**P**,**P’**) Wing discs expressing *mRNA-cap* RNAi and *HA-mRNA-cap* with *MS1096* were immunostained to show the expression of Ci and *ptc-*lacZ. The reduced expression of Ci and *ptc-*lacZ were rescued when co-expression of *HA-mRNA-cap* (compare **P**,**P’** with **M** and **J**). All images are representative of three independent experiments.

Since *mRNA-cap* knockdown disrupts the wing morphology and many signaling pathways including Hh, Wingless, Notch and Hippo pathways are involved in the regulation of the wing development, we tested whether *mRNA-cap* knockdown affects these pathways. We found that Wingless, Notch and Hippo signaling pathway activities exhibited little if any changes when *mRNA-cap* was knocked down, as monitored by their cognate read-outs *Dll-*lacZ (Fig. 2A,B), Cut (Fig. 2C,D) and *Ex*-lacZ (Fig. 2E,F) respectively. However, we found that *mRNA-cap* RNAi downregulated the expression of Hh pathway target genes including *dpp*-lacZ, *ptc-*lacZ and En (Fig. 2H,J and L compared with Fig. 2G’,I’ and K’), suggesting that mRNA-cap selectively modulates Hh signaling activity.

In order to future test the function of *mRNA-cap* in the Hh pathway, we detected the Ci and found that the level of Ci^F^ was downregulated by *mRNA-cap* RNAi (Fig. 2M). We obtained similar results using an independent RNAi line (32847) targeting *mRNA-cap* (Supplementary Fig. 1C–C”’). To confirm the function of mRNA-cap in the Hh pathway, we employed the FLP-FRT mitotic recombination technique to generate homozygous clones for an *mRNA-cap* mutant, *mRNA-cap* ^*A*^. Consistent with the observation that *mRNA-cap* RNAi downregulated Ci^F^ level, *mRNA-cap* ^*A*^ clones, which are marked by lack of GFP expression (Fig. 2N,O), showed decreased Ci^F^ level (Fig. 2N’,O’). Furthermore, co-expression of *HA-mRNA-cap* with *mRNA-cap* RNAi rescued the expression of Ci^F^ and *ptc*-lacZ (Fig. 2P,P’). These observations indicate that mRNA-cap exerts a positive influence on Hh pathway activity.

### Knockdown of mRNA-cap downregulates Ci but upregulates Smo

**Figure 3:**
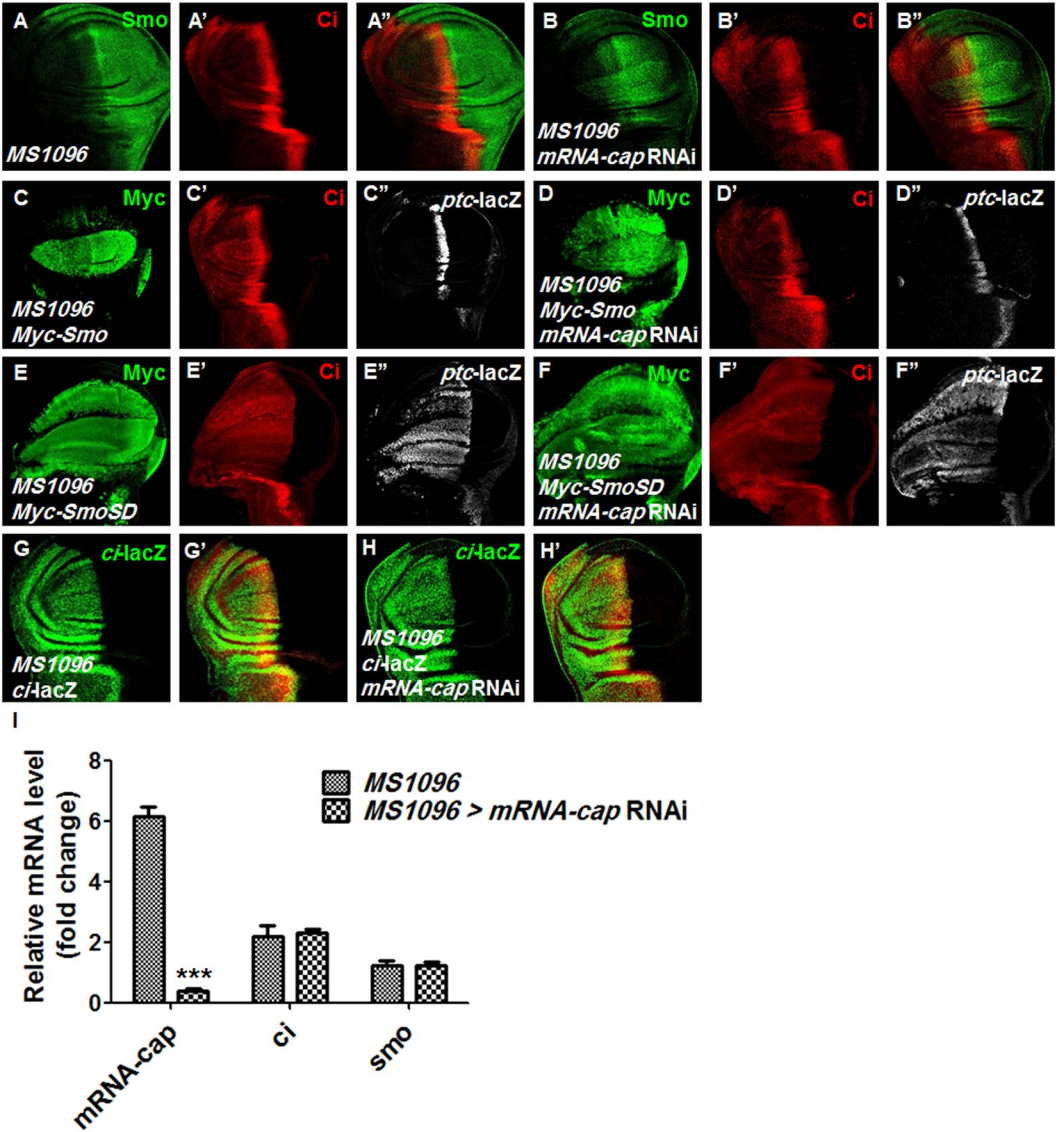
Knockdown of *mRNA-cap* upregulates Smo while downregulates Ci levels. (**A**–**A”**) Control wing discs carrying *MS1096* Gal4 driver were stained to show endogenous Smo (green) and full-length Ci (red). (**B**–**B”**) Wing discs expressing *mRNA-cap* RNAi with *MS1096* were immunostained with Smo (green) and Ci (red) antibodies. Smo was accumulated in A and P compartments when *mRNA-cap* was knocked down. (**C**–**C”**) Wing discs expressing *Myc-Smo* with *MS1096* were immunostained to show the expression of Myc, Ci and *ptc*-lacZ. (**D**–**D”**) Wing discs expressing *Myc-Smo* and *mRNA-cap* RNAi with *MS1096* were stained to show the expression of Myc, Ci and *ptc*-lacZ. Ci and *ptc*-lacZ were also decreased when overexpressing Smo and *mRNA-cap* RNAi. (**E**–**E”**) Wing discs expressing *Myc-SmoSD* with *MS1096* were immunostained to show the expression of Myc, Ci and *ptc*-lacZ. (**F**–**F”**) Wing discs expressing *Myc-SmoSD* with *mRNA-cap* RNAi by *MS1096* were stained for Myc, Ci and *ptc*-lacZ. Ci and *ptc*-lacZ were also decreased when overexpressing activated Smo and *mRNA-cap* RNAi. (**G**,**G’**,**H**,**H’**) Wing discs expressing *mRNA-cap* RNAi and *ci-*lacZ with *MS1096* were immunostained to show the expression of *ci*-lacZ. The expression of *ci*-lacZ alone was used as a control. (**I**) The relative mRNA levels of *ci* and *smo* of wing discs measured by RT-qPCR in *mRNA-cap* knockdown background. *MS1096* wing discs were used as a control. All images are representative of three independent experiments. Data presented are the average of three independent experiments and error bars represent SD. ^***^P < 0.001, t-test.

From the above results, we found that knockdown of *mRNA-cap* led to a decrease of Ci^F^ level, which is consistent with the observed downregulation of Hh pathway target genes expression. However, when we stained the wing discs with a Smo antibody, we found unexpectedly that knockdown of *mRNA-cap* resulted in Smo accumulation in A cells distant from the A/P boundary (Fig. 3B compared with Fig. 3A) even though Ci was downregulated (Fig. 3A’,B’), suggesting that mRNA-cap may regulate both Smo and Ci level but in the opposite direction.

To further confirm this notion, we co-overexpressed *mRNA-cap* RNAi with *UAS-SmoWT* or *UAS-SmoSD*, which is a constitutively active form of Smo with PKA sites mutated to amino acids residues Asp, and found that knockdown of *mRNA-cap* downregulated Ci (Fig. 3 compare C’,E’ to D’,F’) and *ptc*-lacZ (Fig. 3 compare C’’,E’’ to D’’,F’’) even in the presence of Smo overexpression, indicating that *mRNA-cap* RNAi can block the Hh signal transduction at a step downstream of Smo.

To distinguish whether Ci downregulation by *mRNA-cap* RNAi occurs at mRNA or protein level, we examined the transcript level of *ci* using the *ci*-lacZ reporter that expresses lacZ under the control of *ci* promotor. We didn’t find any difference in *ci*-lacZ expression between the control and *mRNA-cap* knockdown wing discs (Fig. 3G–H’), indicating that *ci* transcription is not regulated by *mRNA-cap*. To exclude that mRNA-cap affects Ci mRNA stability, we measured *ci* mRNA level by RT-qPCR in wing discs and found that *mRNA-cap* RNAi didn’t affect *ci* mRNA level (Fig. 3I). Meanwhile we found that mRNA-cap did not affect the *smo* mRNA level (Fig. 3I). Taken together, the RT-qPCR results imply that mRNA-cap affects Ci and Smo at protein level instead of mRNA level.

### mRNA-cap regulates Hh pathway activity through PKA

**Figure 4:**
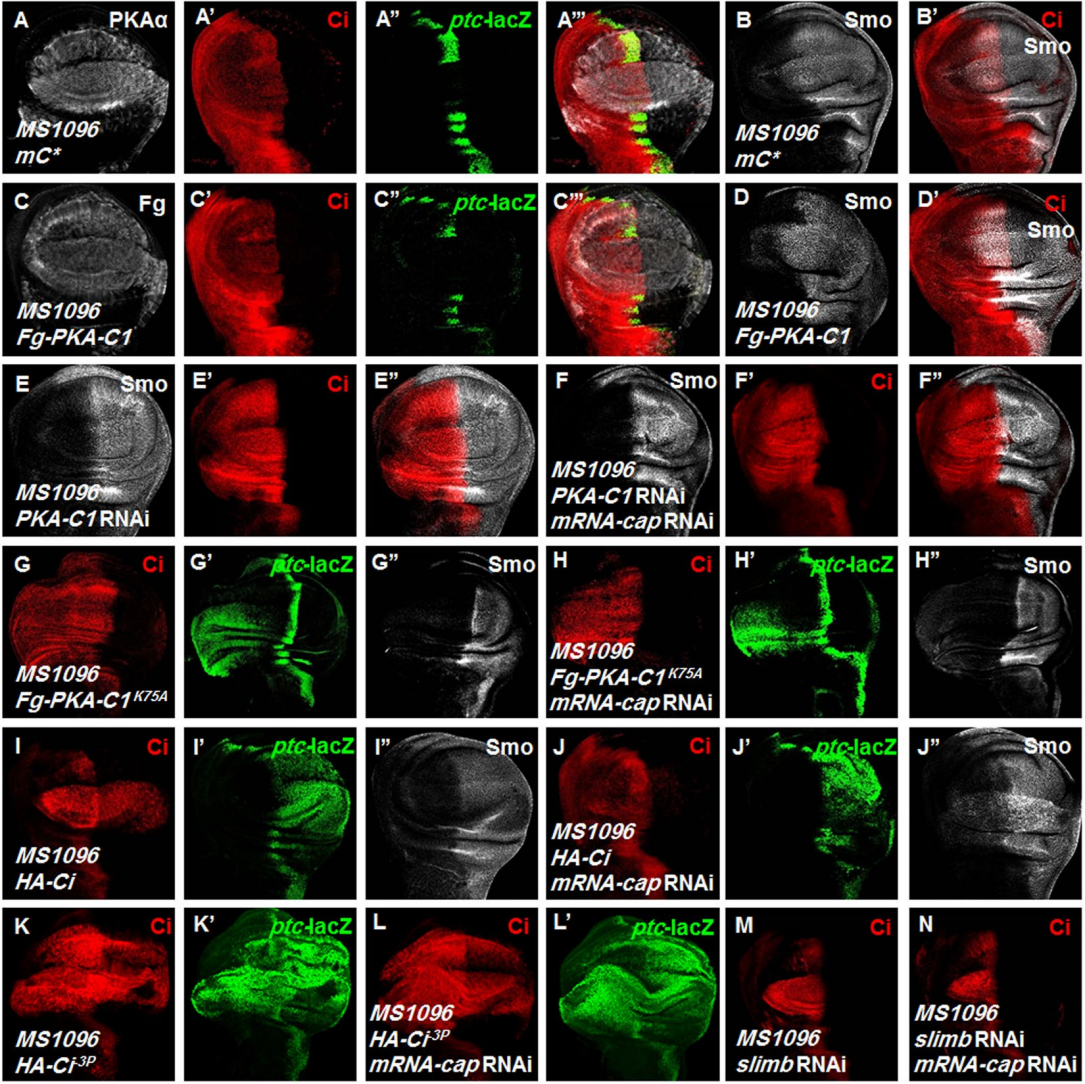
mRNA-cap regulates Hh signaling activity through PKA. (**A**–**B’**) A wing disc expressing mC* with *MS1096* was immunostained to show PKAα, Ci, *ptc*-lacZ and Smo expression. Ci and *ptc*-lacZ levels were downregulated by mC*. However, Smo level was increased. PKAα antibody was used to show mC* expression. (**C**–**D’**) A wing disc expressing *Fg-PKA-C1* with *MS1096* was immunostained to show Fg, Ci, *ptc*-lacZ and Smo. Ci and *ptc*-lacZ levels were downregulated by expression of *Fg-PKA-C1*. But Smo level was up-regulated. (**E**–**F”**) Wing discs expressing *PKA-C1* RNAi alone (**E**–**E”**) or together with *mRNA-cap* RNAi (**F**–**F”**) with *MS1096* were stained for Smo and Ci. The accumulation of Smo and the attenuation of Ci expression caused by *mRNA-cap* knockdown were blocked by *PKA-C1* RNAi. (**G**–**H”**) Wing discs expressing *Fg-PKA-C1* ^*K75A*^ alone or together with *mRNA-cap* RNAi using *MS1096* were immunostained to show Ci, *ptc*-lacZ and Smo expression. (**I**–**J”**) Wing discs expressing *HA-Ci* alone or together with *mRNA-cap* RNAi with *MS1096* were immunostained to show Ci, *ptc*-lacZ and Smo expression. Ci level was unstable upon *mRNA-cap* knockdown. (**K**–**L’**) Wing discs expressing *HA-Ci* ^*−3P*^ alone or together with *mRNA-cap* RNAi using *MS1096* were immunostained to show Ci and *ptc*-lacZ expression. The Ci^−*3P*^ level was stable even *mRNA-cap* was knocked down. (**M**,**N**) Wing discs expressing *slimb* RNAi alone or together with *mRNA-cap* RNAi using *MS1096* were immunostained to show Ci expression. All images are representative of three independent experiments.

Knockdown of *mRNA-cap* causes accumulation of Smo but downregulation of Ci and Hh target genes expression, which is reminiscent of the phenotype caused by excessive PKA activity on Hh signaling^51, 52^. As shown in Fig. 4A–B’, overexpression of mC*, a constitutively active form of mammalian PKA catalytic subunit, resulted in upregulation of Smo but downregulation of Ci and *ptc*-lacZ expression. We obtained similar results when overexpressing the *Drosophila* PKA catalytic subunit 1 (PKA-C1) (Fig. 4C–D’).

To test the genetic interaction between *mRNA-cap* and *PKA*, we expressed *mRNA-cap* RNAi and *PKA-C1* RNAi (VDRC 101524) transgenes individually or in combination. As shown in Fig. 4, we observed that knockdown of *PKA-C1* either alone or in conjunction with *mRNA-cap* resulted in the accumulation of full-length Ci in anterior compartment and prevented the up-regulation of Smo caused by *mRNA-cap* RNAi (Fig. 4E–F”). Furthermore, we employed a dominant negative form of *PKA-C1* (*PKA-C1* ^*K75A*^) with K75 mutated to alanine (A) and obtained results similar to *PKA-C1* RNAi, i.e., Ci and *ptc*-lacZ level were high in anterior compartment but Smo level had no obvious change when *Fg-PKA-C1* ^*K75A*^ was expressed alone (Fig. 4G–G”), and simultaneous overexpression of *Fg-PKA-C1* ^*K75A*^ and *mRNA-cap* RNAi prevented the up-regulation of Smo caused by *mRNA-cap* RNAi but resulted in upregulation of Ci similar to expressing *Fg-PKA-C1* ^*K75A*^ alone (Fig. 4H–H”). This epistasis analysis indicates that mRNA-cap plays the role on upstream of PKA.

To further test this idea, we overexpressed Ci^WT^ or Ci^−3p^ (a constitutively active form of Ci with three PKA sites mutated to Ala) with *mRNA-cap* RNAi and found that mutating the PKA sites in Ci renders it resistant to *mRNA-cap* RNAi-mediated downregulation (Fig. 4I–L’). Phosphorylation of Ci by PKA targets it for Slimb-mediated degradation^24, 53^. We found that knockdown of *slimb* either alone or together with *mRNA-cap* upregulated Ci (Fig. 4M,N). Taken together, these results suggest that mRNA-cap regulates Smo and Ci through PKA.

### mRNA-cap inhibits the PKA activity in wing discs

**Figure 5:**
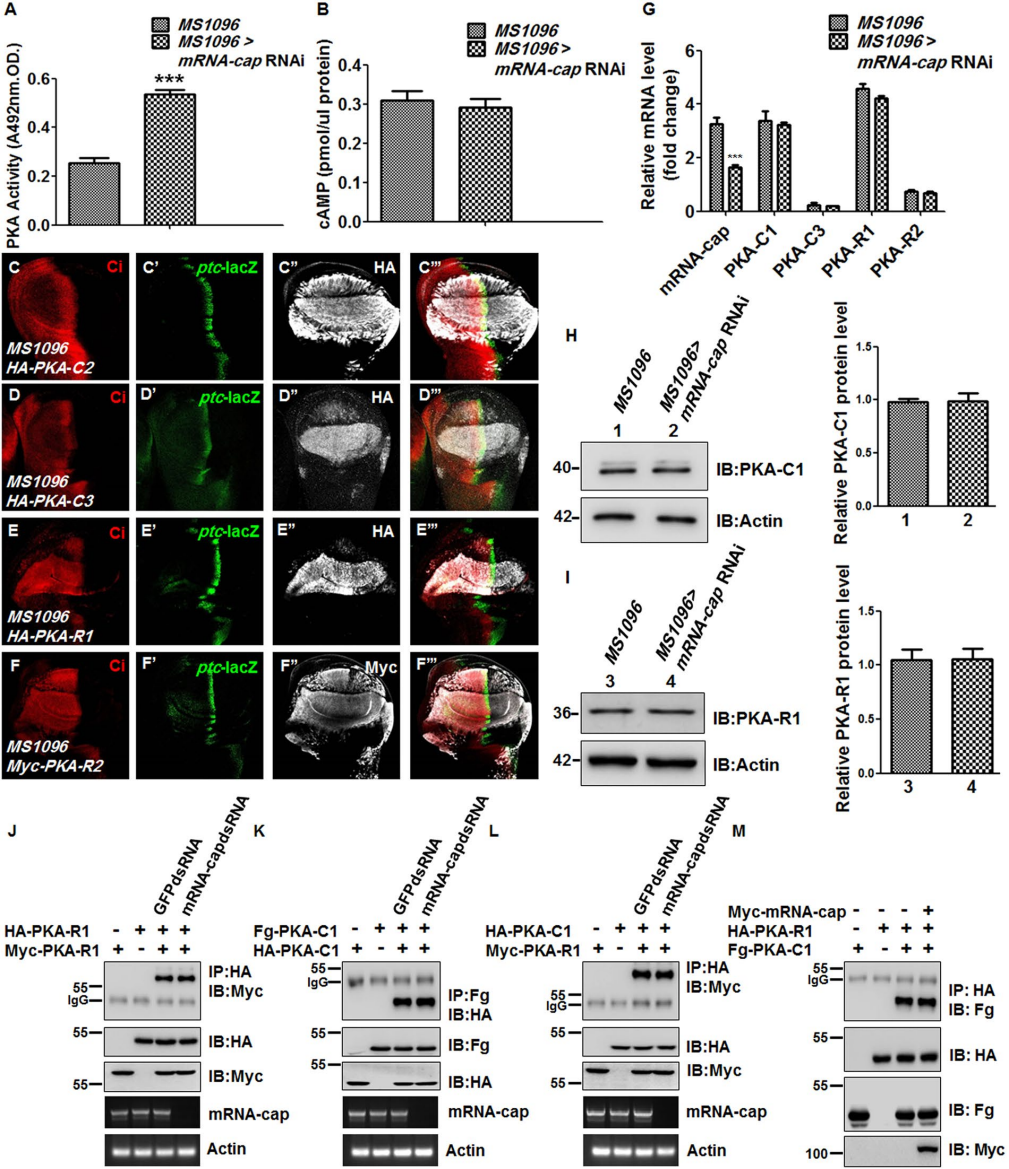
mRNA-cap regulates PKA activity. (**A**,**B**) The PKA activity (**A**) and cAMP level (**B**) were measured from control wing discs or wing discs expressing *mRNA-cap* RNAi with *MS1096*. Approximately 400 discs were dissected and lysed for each assay. (**C**–**C”’**) Overexpression of *HA-PKA-C2* by *MS1096* was immunostained for Ci, *ptc*-lacZ and HA. (**D**–**D”’**) Overexpression of *HA-PKA-C3* by *MS1096* was immunostained for Ci, *ptc*-lacZ and HA. The levels of Ci were decreased. (**E**–**F”’**) Overexpression of *HA-PKA-R1* (**E**–**E”’**) or *Myc-PKA-R2* (**F**–**F”’**) with *MS1096* was immunostained for Ci, *ptc*-lacZ and HA or Myc. The levels of Ci and *ptc*-lacZ were up-regulated. (**G**) The relative mRNA levels of *PKA-C1*, *PKA-C3*, *PKA-R1* and *PKA-R2* of wing discs were measured by RT-qPCR upon knockdown of *mRNA-cap*. *MS1096* acted as a control. (**H**) Western blot analysis of lysates from control wing discs or wing discs expressing *mRNA-cap* RNAi with *MS1096*. Approximately 100 discs were dissected, lysed, and blotted with anti-PKA-C1 antibody, respectively. (**I**) Western blot analysis of PKA-R1 levels from control wing discs or wing discs expressing *mRNA-cap* RNAi. Approximately 100 discs were used. (**J**,**K**) Knockdown of *mRNA-cap* did not affect the dimer formation of PKA-R1 (**J**) or PKA-C1 (**K**). (**L**,**M**) mRNA-cap didn’t change the interaction between PKA-C1 and PKA-R1. All images are representative of three independent experiments. (**A**,**B**,**G**,**H** and **I**) Data presented are the average of three independent experiments and error bars represent SD. ^***^P < 0.001, t-test.

The above genetic evidence suggests that mRNA-cap regulates Hh signaling activity through PKA. Next, we tested whether PKA activity is altered upon knockdown of *mRNA-cap*. We found that the PKA activity was increased by *mRNA-cap* RNAi in wing discs (Fig. 5A). As PKA activity is regulated by cAMP, we further tested whether mRNA-cap regulated PKA activity through cAMP and found that cAMP level was not changed when *mRNA-cap* was knocked down (Fig. 5B).

In *Drosophila*, there are two PKA regulatory subunits: PKA-R1, R2 and three PKA catalytic subunits: PKA-C1, C2 and C3. We generated transgenic flies expressing these PKA subunits under the control of *UAS* promoter. Except for PKA-C2, over-expression of all other transgenes had the expected effects on Hh signaling; however, the effect of PKA-C3 overexpression on Hh signaling was relatively weak compared with PKA-C1 (Fig. 5C–F”’). We further examined the endogenous mRNA levels of these PKA subunits upon knockdown of *mRNA-cap*, and found no obvious changes despite of efficient knockdown of *mRNA-cap* (Fig. 5G).

As PKA-C1 and PKA-R1 are predominantly expressed in wing discs compared with other PKA subunits (Fig. 5G), their protein levels were further investigated. Comparing the *MS1096* > *mRNA-cap* RNAi with control wing discs, we found that neither PKA-C1 nor PKA-R1 exhibited significant changes in their protein levels (Fig. 5H,I). Taken together, these results suggest that mRNA-cap regulates PKA activity independent of cAMP and the protein levels of PKA regulatory and catalytic subunits.

We next examined whether *mRNA-cap* affects the interaction between the regulatory and catalytic subunits and found that mRNA-cap didn’t affect R1/R1, C1/C1, C1/R1 dimer formation (Fig. 5J–M; Supplementary Fig. 2A–C). All these results indicate that mRNA-cap may regulate PKA activity through a non-canonical mechanism.

### mRNA-cap regulates Hh signaling through its enzyme activity

**Figure 6:**
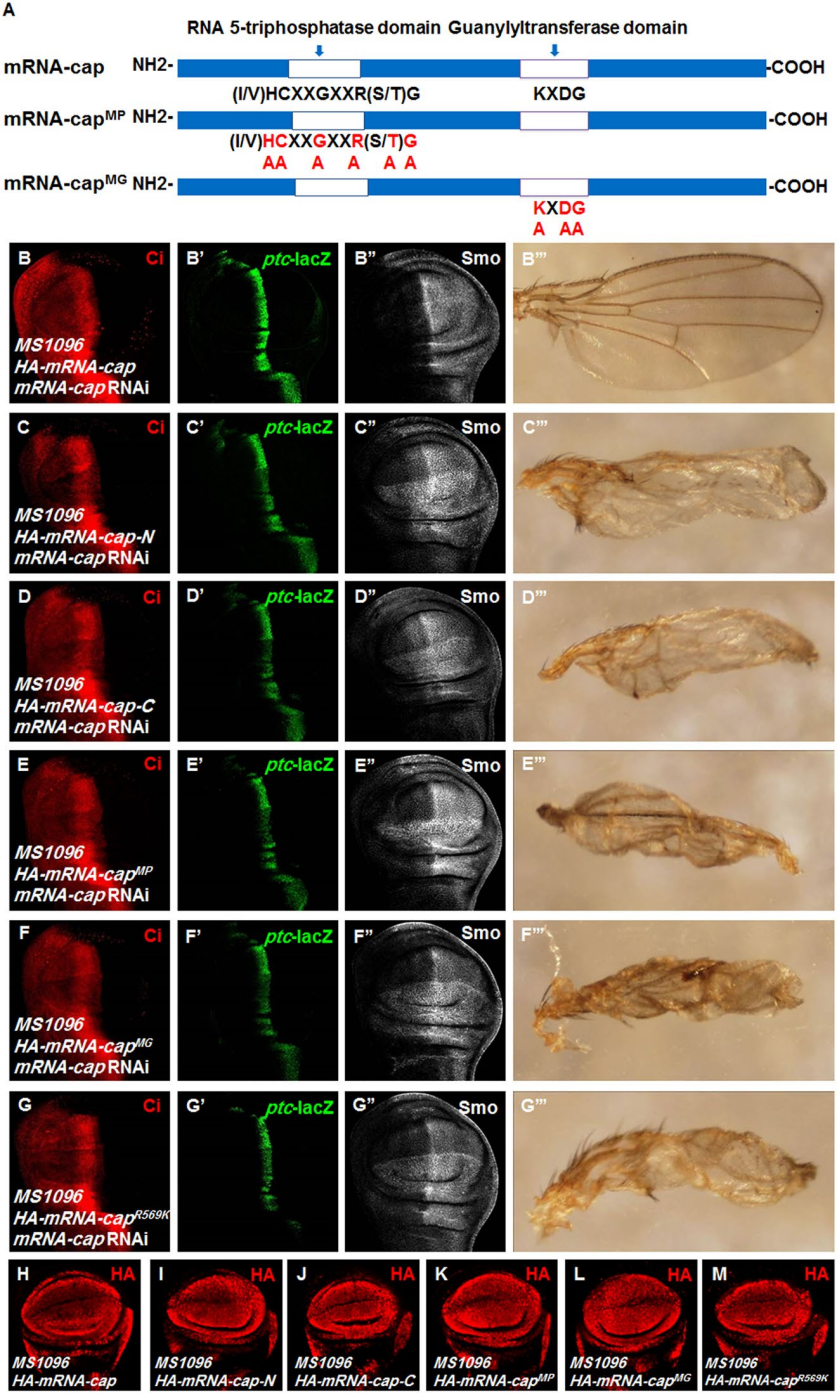
Regulation of Hh signaling by mRNA-cap depends on its enzymatic activity. (**A**) Schematic drawings show the domains and motifs in wild type mRNA-cap and its mutant forms used in transgenic flies and immunofluorescence assays. White bars denoted the RNA 5′-triphosphatase and 3′-guanylyltransferase domains. (**B**–**B”’**) Wing discs expressing *HA-mRNA-cap* and *mRNA-cap* RNAi were stained for Ci, *ptc*-lacZ and Smo. The attenuation of Ci and *ptc*-lacZ caused by *mRNA-cap* knockdown was restored by the expression of *HA-mRNA-cap*. Meanwhile, the accumulation of Smo was suppressed. Adult wing phenotype was also rescued by the expression of *HA-mRNA-cap*. (**C**–**D”’**) Wing discs expressing *HA-mRNA-cap-N* or *HA-mRNA-cap-C* with *mRNA-cap* RNAi were stained for Ci, *ptc*-lacZ and Smo. Neither the defects in Ci, *ptc*-lacZ and Smo expression nor the adult wing phenotypes were rescued. (**E**–**F”’**) Wing discs expressing *HA-mRNA-cap* ^*MP*^ or *HA-mRNA-cap* ^*MG*^ with *mRNA-cap* RNAi were stained for Ci, *ptc*-lacZ and Smo. The Ci, *ptc*-lacZ, Smo and adult wing phenotypes could not be restored. (**G**–**G”’**) Wing discs expressing *HA-mRNA-cap* ^*R569K*^ with *mRNA-cap* RNAi were stained for Ci, *ptc*-lacZ and Smo. Ci, *ptc*-lacZ, Smo and adult wing phenotype also could not be rescued. (**H**–**M**) The expression levels of *HA-mRNA-cap* (**H**), *HA-mRNA-cap*-N (**I**), *HA-mRNA-cap*-C (**J**), *HA-mRNA-cap* ^*MP*^ (**K**), *HA-mRNA-cap* ^*MG*^ (**L**) and *HA-mRNA-cap* ^*R569K*^ (**M**) were comparable in the wing discs expressing these transgenes with *MS1096*. All images are representative of three independent experiments.

To test which domain of mRNA-cap is essential for its regulation of Hh signaling, we generated two transgenes: *HA-mRNA-cap-N* (aa1-272) and *HA-mRNA-cap-C* (aa248-616), which harbor the N-terminal 5′-triphosphatase domain and the C-terminal guanylyltransferase domain, respectively. We co-expressed *mRNA-cap* RNAi with *HA-mRNA-cap*, *HA-mRNA-cap-N* or *HA-mRNA-cap-C* and found that only *HA-mRNA-cap* rescued the adult wing phenotype (Fig. 6B”’) as well as the expression of Ci, *ptc*-lacZ and Smo to wild type levels (Fig. 6B–B”), whereas neither *HA-mRNA-cap-N* nor *HA-mRNA-cap-C* could rescue the RNAi phenotypes (Fig. 6C–D”’), indicating that both N- and C-terminus of *mRNA-cap* are essential for its function in the Hh signaling pathway.

We next generated transgenic flies expressing two mutant forms of *mRNA-cap, mRNA-cap* ^*MP*^ and *mRNA-cap* ^*MG*^, which mutated the essential residues in the triphosphatase and guanylyltransferase domains, respectively (Fig. 6A)^48^. We found that neither *mRNA-cap* ^*MP*^ nor *mRNA-cap* ^*MG*^ could rescue the abnormal wing phenotypes as well as the abnormal expression of Ci, *ptc*-lacZ and Smo caused by *mRNA-cap* RNAi (Fig. 6E–F”’). These results suggest that modulation of Hh signaling by *mRNA-cap* may depend on its capping enzyme activity. This notion was further supported by the fact that a single amino acid substitution in *mRNA-cap*, *mRNA-cap* ^*R569K*^, which affects its interaction with poly (U) and disrupts the capping enzyme activity in mammalian^48^, affected its ability to rescue Hh signaling defects caused by *mRNA-cap* RNAi (Fig. 6G–M). Taken together, these results suggest that mRNA-cap regulates Hh signaling activity depending on its capping enzyme activity.

**Figure 7:**
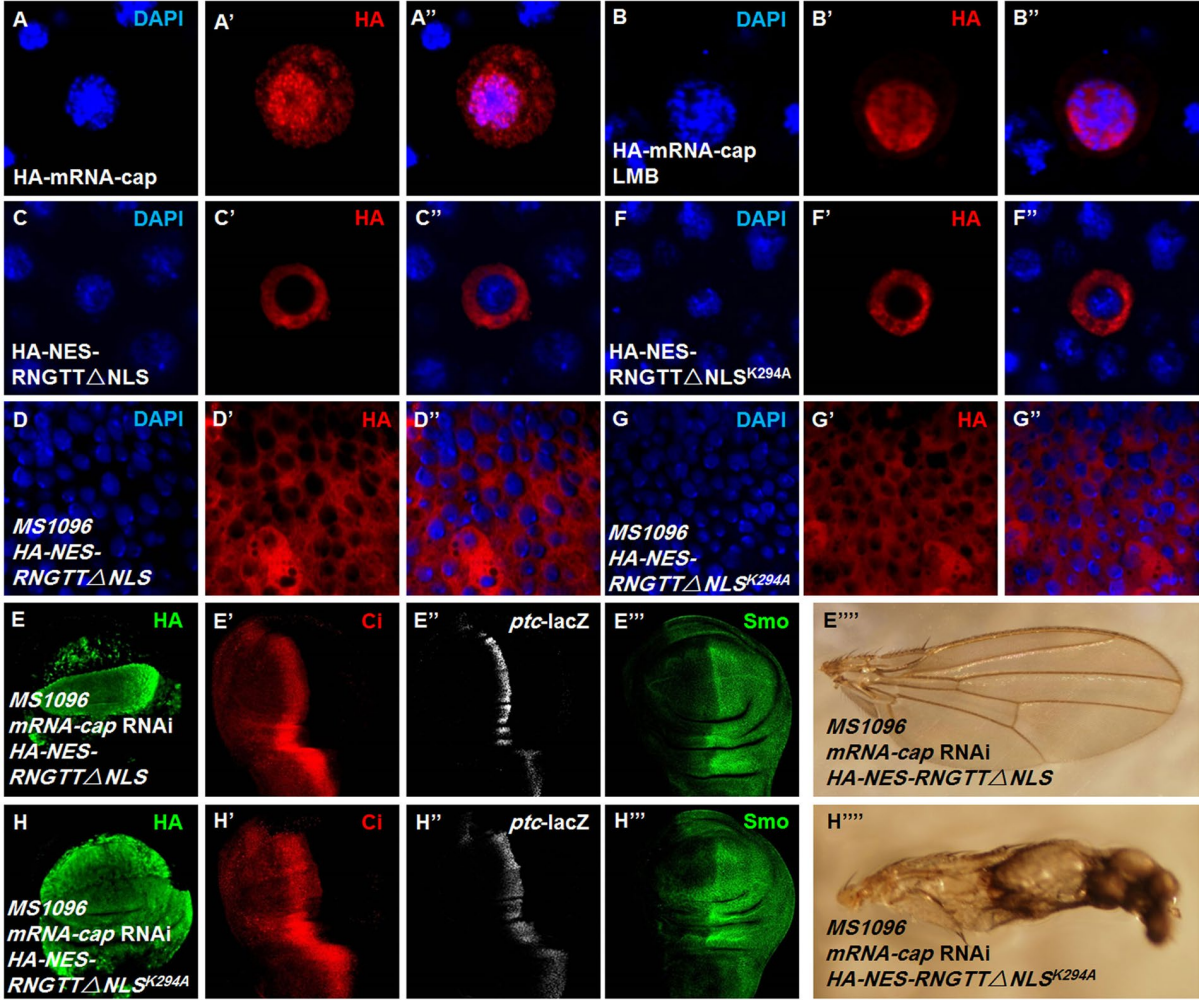
Cytoplasmic *mRNA-cap* regulates the Hh pathway. (**A**–**A”**) S2 cells expressing HA-mRNA-cap were stained to show DAPI and HA. (**B**–**B”**) LMB treatment resulted in nuclear localization of HA-mRNA-cap in S2 cells. (**C**–**D”**) S2 cells and wing discs expressing HA-NES-RNGTT△NLS were stained for DAPI and HA. (**E**–**E””**) Wing discs expressing *HA-NES-RNGTT* △*NLS* with *mRNA-cap* RNAi were stained for HA, Ci, *ptc*-lacZ and Smo. The detects in Ci, *ptc*-lacZ and Smo levels as well as adult wing phenotype were rescued. (**F**–**G”**) S2 cells and wing discs expressing HA-NES-RNGTT△NLS^K294A^ were stained for DAPI and HA. (**H**–**H””**) Wing discs expressing *HA-NES-RNGTT*△*NLS* ^*K294A*^ under knockdown of *mRNA-cap* background were stained for HA, Ci, *ptc*-lacZ and Smo. The defects in Ci, *ptc*-lacZ, Smo levels and adult wing phenotype could not be rescued. All images are representative of three independent experiments.

To define where mRNA-cap exerts its influence on Hh signaling, we transfected HA-mRNA-cap in S2 cells and found that it is located in both cytoplasm and nucleus (Fig. 7A–A”). Treating the cells with the nuclear export inhibitor LMB resulted in an exclusive nuclear localization of HA-mRNA-cap (Fig. 7B–B”), suggesting that mRNA-cap shuttles between the nucleus and cytoplasm and may modulate PKA activity in the cytoplasm, nucleus or both.

To determine where mRNA-cap acts, we overexpressed an mRNA-cap variant, HA-NES-RNGTT△NLS, which is exclusively localized in the cytoplasm both in the S2 cells and in the wing discs (Fig. 7C–D”), and found that it could rescue the Ci, *ptc*-lacZ, Smo levels (Fig. 7E–E”’) as well as adult wing phenotype in *mRNA-cap* RNAi background (Fig. 7E””). On the other hand, a capping enzyme-dead cytoplasmic form of mRNA-cap, HA-NES-RNGTT△NLS^K294A^ (Fig. 7F–G”) failed to rescue the Hh pathway defects caused by *mRNA-cap* RNAi (Fig. 7H–H””). These results suggest that mRNA-cap can regulate Hh signaling in the cytoplasm through its capping enzyme activity.

### Mammalian mRNA-cap is functionally conserved

**Figure 8:**
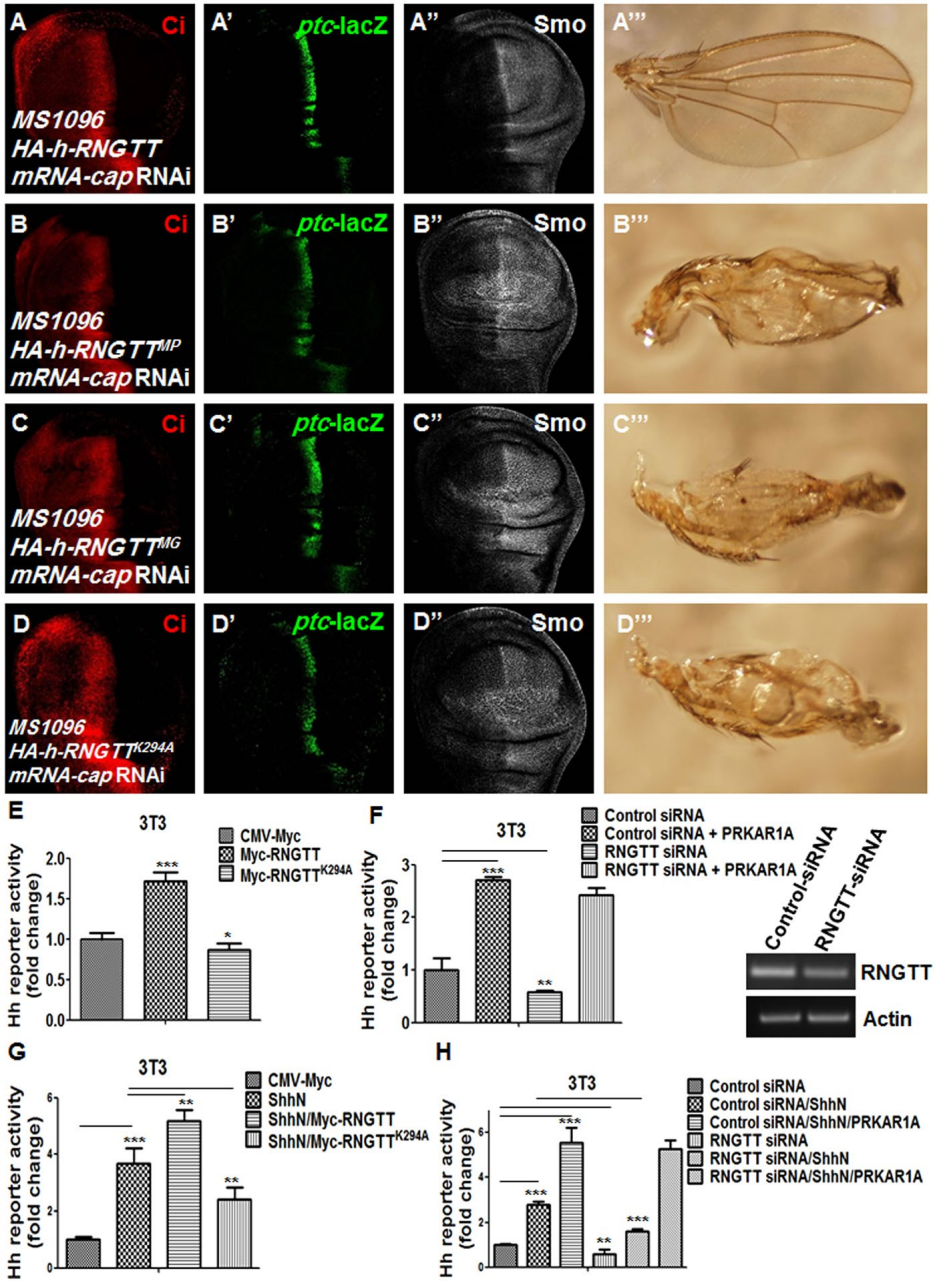
A conserved function of human mRNA-cap in Hh pathway regulation. (**A**–**A”**) A wing disc expressing *HA-h-RNGTT* and *mRNA-cap* RNAi with *MS1096* was immunostained for Ci, *ptc*-lacZ and Smo. Defects in Ci, *ptc*-lacZ and Smo level were rescued by *HA-h-RNGTT* overexpression. (**A”’**,**B”’**,**C”’**,**D”’**) Adult wings expressing *HA-h-RNGTT* (**A”’**), *HA-h-RNGTT* ^*MP*^ (**B”’**), *HA-h-RNGTT* ^*MG*^ (**C”’**) or *HA-h-RNGTT* ^*K294A*^ (**D”’**) in *mRNA-cap* knockdown background. Only expression of *HA-h-RNGTT* could rescue the adult wing defect induced by *mRNA-cap* RNAi. (**B**–**D”**) Wing discs expressing *mRNA-cap* RNAi in conjunction with *HA-h-RNGTT* ^*MP*^ (**B**–**B”**) or *HA-h-RNGTT* ^*MG*^ (**C**–**C”**) or *HA-h-RNGTT* ^*K294A*^ (**D**–**D”**) were immunostained for Ci, *ptc*-lacZ and Smo. The defects in Ci, *ptc*-lacZ and Smo levels were not rescued by the expression of *HA-h-RNGTT* ^*MP*^, *HA-h-RNGTT* ^*MG*^ or *HA-h-RNGTT* ^*K294A*^. (**E**–**H**) *Gli-luciferase* (*Gli-luc*) reporter assays in NIH/3T3 cells transfected with the indicated constructs in the absence (**E**,**F**) or presence (**G**,**H**) of Shh treatment. Gli luciferase activities were normalized to Renilla luciferase activities. RNGTT increased whereas RNGTT^K294A^ or knockdown of *RNGTT* decreased Shh pathway activity. Co-transfection with PRKAR1A restored the pathway activity. The knockdown efficiency of *RNGTT* was assessed through PCR. Actin acts as a loading control. All images are representative of three independent experiments. Data presented are the average of three independent experiments and error bars represent SD. ^*^P < 0.05, ^**^P < 0.01, ^***^P < 0.001, t-test.

Our above results show mRNA-cap regulates Hh signaling activity. To test whether mammalian mRNA-cap is functionally conserved throughout evolution, we sub-cloned *RNGTT*, the homolog of *mRNA-cap* in humans, into the *UAS* vector and generated transgenic flies carrying *UAS-RNGTT*. Co-expression of *UAS-RNGTT* with the *mRNA-cap* RNAi transgene rescued the wing phenotype and restored the normal levels of Ci, *ptc*-lacZ and Smo (Fig. 8A–A”’), suggesting that RNGTT could substitute *Drosophila mRNA-cap* to regulate Hh signaling, indicating that *mRNA-cap* is functionally conserved between *Drosophila* and mammals. Furthermore, mutating the essential residues in the 5′-triphosphatase (MP) or the 3′-guanylyltransferase (MG) domain of human RNGTT or a single point mutation (K294A) in RNGTT, which eliminated its guanylylation function^54^, all prevented it from rescuing the Hh signaling defects and adult wing phenotypes caused by *mRNA-cap* RNAi (Fig. 8B–D”’). Taken together, the results suggest that RNGTT is functionally conserved during evolution, and it can substitute *mRNA-cap* to modulate Hh singling depending on the capping enzyme activity.

To further confirm the conservation of mRNA-cap function in the Hh signaling pathway, we performed the *Gli*-*luciferase* reporter assay in NIH/3T3 cells, which was regulated by Sonic hedgehog (Shh) pathway components. Results from the reporter assay showed that overexpression of RNGTT promoted *Gli-luciferase* activity whereas RNGTT^K294A^ decreased the Hh pathway activity (Fig. 8E). Consistently, knockdown of endogenous *RNGTT* suppressed *Gli-luciferase* activity (Fig. 8F), indicating that RNGTT is a functionally conserved positive regulator in the Shh pathway. Meanwhile, we found that expression of the mammalian homolog of PKA-R1, PRKAR1A, increased the expression of *Gli-luciferase* reporter gene in both control and *RNGTT* knockdown cells (Fig. 8F). We obtained the similar results when cells were treated with ShhN (Fig 8G,H). Taken together, these results support that *RNGTT* regulates Shh pathway activity through PKA.

## Discussion

**Figure 9:**
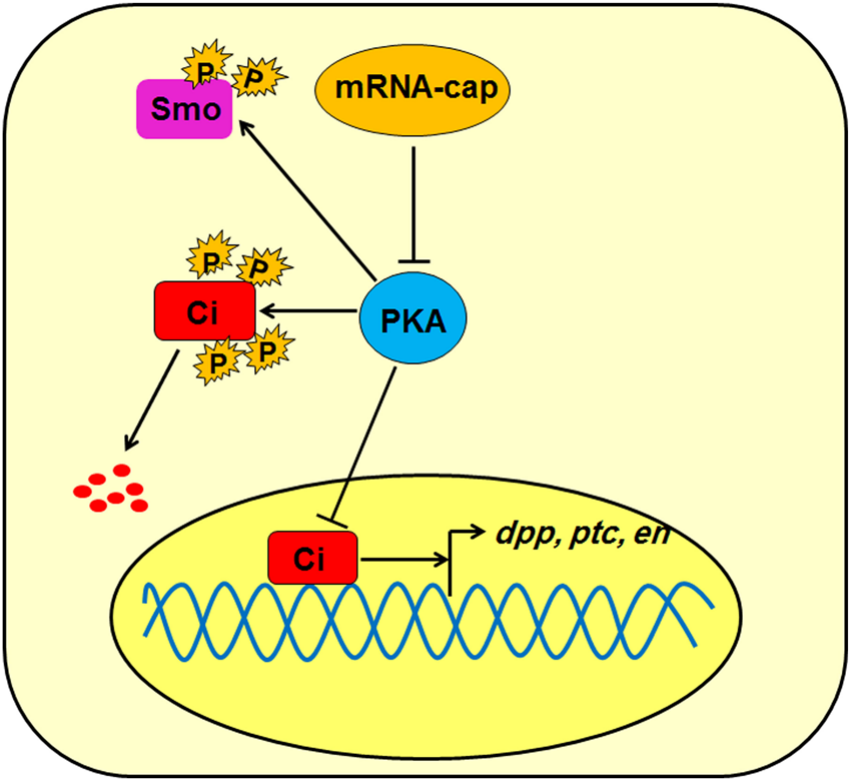
Regulation of Hh signaling through the mRNA-cap-PKA axis. mRNA-cap inhibits PKA activity to modulate the stability of Ci and Smo, thus affecting Hh pathway output.

Hh signaling plays essential roles in embryonic development and adult tissue homeostasis, and its misregulation causes numerous human diseases including birth defects and cancers. PKA modulates Hh signaling activity through phosphorylation of the transcription factor Ci/Gli and the GPCR family protein and Hh signal transducer Smo. Besides the regulation of PKA-substrate interaction^55^ and possible involvement of Gαi^56^, how PKA activity is regulated remains poorly understood. In this study, we identify a novel regulator of the Hh pathway, the capping-enzyme mRNA-cap, which positively regulates Hh signaling activity through modulating PKA activity (Fig. 9). Interestingly, we demonstrate that the regulation of Hh signaling by *mRNA-cap* can be achieved by its cytoplasmic capping-enzyme activity. Finally, we show that the mammalian homolog of *mRNA-cap*, RNGTT, plays a conserved role in the regulation of Hh signaling. Interestingly, it has been reported recently that mRNA capping machinery can regulate specific gene expression, and is involved in the maintenance of pluripotency and differentiation of embryonic stem cell (ESC), the regulation of G1 Phase transcripts, and the regulation of the Wnt/β-catenin signaling activity^57–59^. Here, our study links the mRNA capping machinery to another important developmental signaling pathway, Hh signaling pathway, unveils a new facet of Hh signaling regulation and uncovers a potential new target for modulating Hh signaling activity in the treatment of cancer.

As shown in Flybase, *Drosophila* has only one capping enzyme encoded by *mRNA-cap*, which harbors an RNA 5′-triphosphatase domain and an mRNA 3′-guanylyltransferase domain and catalyzes the attachment of the 5′ cap to messenger RNA molecules in the nucleus during the first stage of gene expression. Ubiquitous knockdown of *mRNA-cap* causes fly lethality. In addition, *in situ* hybridization assay reveals that its expression is ubiquitous in *Drosophila* wing discs (Supplementary Fig. 1D,D’). These observations suggest that *mRNA-cap* is an essential gene for fly development. Surprisingly, knockdown of *mRNA-cap* can selectively affect Hh signaling without affecting several other signaling pathways we have tested. Our results indicate that mRNA-cap shuttles between the cytoplasm and the nucleus, implying that mRNA-cap may also play a role in the cytoplasm in addition to its well-characterized function in nucleus. As mentioned before, mRNA-cap can recap a selective fraction of translationally inactive mRNAs to return them to an active state through cytoplasmic re-capping mechanism^50^. In this case, there may be only a small number of genes that are controlled by mRNA-cap through cytoplasmic capping function, one of which could be involved in regulating PKA and Hh signaling. As shown in Fig. 7 and Supplementary Fig. 3A,B, the results in both *Drosophila* and the NIH/3T3 cells support this idea.

How mRNA-cap affects PKA activity is not resolved and remains an important issue to address by future study. The PKA holoenzyme consists of two catalytic (C) subunits and two regulatory (R) subunits, cAMP binding to R subunit and the subsequent dissociation between R and C subunits are needed for PKA activity. Our results indicate that knockdown of *mRNA-cap* upregulated PKA activity, but did not affect cAMP level, *PKA-R1*, *R2*, *C1*, *C3* mRNA levels and R1, C1 protein levels. Neither did it affect PKA-R1/R1, C1/C1 and R1/C1 interaction. In addition, the regulation of Hh signaling by *mRNA-cap* is dependent on its cytoplasmic capping-enzyme activity, and knockdown of the *mRNA cap* binding protein, Cbp80, showed similar Hh signaling defects (Supplementary Fig. 4A–C), indicating that the mRNA capping machinery is involved in the regulation of Hh signaling activity. Since PKA can directly bind Smo and Cos2^36, 55^, we also carried out experiments to exclude the possibility that mRNA-cap regulates the interactions of Cos2-PKA and Smo-PKA (Supplementary Fig. 5A,B). Taken together, our results suggest that mRNA-cap may regulate an unidentified factor that is involved in the inhibition of PKA activity through a non-canonical mechanism.

Importantly, our results indicate that mRNA-cap function is conserved during evolution. Given that PKA plays essential roles in developmental and adult homeostasis as well as a variety of physiological conditions, our study suggests an alternative way to modulate PKA activity and indicates that mRNA-cap and its relevant target genes may be the potential drug targets for the treatment of Hh- or PKA-related diseases.

## Materials and Methods

### Constructs, Mutants and Transgenes

The constructs for S2 cell transfection experiments are as follows: Myc/Fg/HA-mRNA-cap, Fg/HA-PKA-C1, Myc/HA-PKA-R1, Myc-Cos2 and Myc-Smo, the corresponding cDNA fragments of which were amplified, and cloned into the pUAST vectors. Similarly, the constructs of Myc-RNGTT, Myc-RNGTT^K294A^, Myc-PRKAR1A, HA-NES-RNGTT△NLS and HA-NES-RNGTT△NLS^K294A^ used in mammal cells were cloned into the pcDNA3.1 vectors. The mutants of mRNA-cap/RNGTT, including HA-mRNA-cap^MP^, HA-mRNA-cap^MG^, HA-mRNA-cap-N (aa1-272), HA-mRNA-cap-C (aa248-616), HA-mRNA-cap^R569K^, HA-RNGTT^MP^, HA-RNGTT^MG^ and HA-RNGTT^K294A^ were constructed in the backbones of mRNA-cap or RNAGTT through the deletion of the corresponding regions or PCR-based site-directed mutagenesis. We also added the nuclear export signal (NES) LQLPPLERLTLD and deleted the nuclear localization signal (NLS) KRKH to generate the HA-NES-RNGTT△NLS and HA-NES-RNGTT△NLS^K294A^ plasmids in the background of RNGTT and RNGTT^K294A^. The RNAi lines that targeted *mRNA-cap* (v108809, BL32847), *PKA-C1* (v101524), *Cbp80* (7035R-3) and *slimb* (3412R-1) were obtained from the Vienna *Drosophila* RNAi Center (VDRC), the Bloomington Stock Center and National Institute of Genetics (NIG), respectively. Flies of *MS1096*, *C765-Smo* ^*-PKA12*^, *Hh-Gal4*, *En-Gal4*, *HA-Ci*, *HA-Ci* ^*−3P*^, *Myc-Smo*, *Myc-SmoSD*, *ptc*-lacZ, *dpp*-lacZ, *Dll-*lacZ, *Ex-*lacZ, *ci*-lacZ, mC*, *Fg-PKA-C1* (BL35554) and *Fg-PKA-C* ^*K75A*^ (35559) have been described (Flybase)^60^. *mRNA-cap* ^*A*^ is an *mRNA-cap* mutant allele, whose tyrosine (Y) at 346 is replaced by alanine (A) (Flybase). The transgenic flies of HA-mRNA-cap, HA-mRNA-cap^MP^, HA-mRNA-cap^MG^, HA-mRNA-cap-N, HA-mRNA-cap-C, HA-mRNA-cap^R569K^, HA-RNGTT, HA-RNGTT^MP^, HA-RNGTT^MG^, HA-RNGTT^K294A^, HA-PKA-C2, HA-PKA-C3, HA-PKA-R1, Myc-PKA-R2, HA-NES-RNGTT△NLS and HA-NES-RNGTT△NLS^K294A^ were generated by injection of corresponding constructs into *Drosophila* embryos according to the methods described previously^61^. The parental strain for all germline transformations was *w* ^*1118*^. All stocks used in this study were maintained and raised under standard conditions.

### Immunostaining and *in situ* hybridization

Immunostaining and *in situ* hybridization of imaginal discs were performed according to the standard protocols^53, 62^. Antibodies were used in this study as follows: rat anti-Ci (2 A) (DSHB, 1:50), mouse anti-Flag (M2) (Sigma,1:200), mouse anti-HA (F7) (Santa Cruz, 1:200), mouse anti-Myc (9E10) (Santa Cruz, 1:5000), mouse anti-β-galactosidase (Sigma, 1:500), mouse anti-En (DSHB, 1:50), mouse anti-Smo (DSHB, 1:50), mouse anti-Cut (DSHB, 1:50), 4,6-iamidino-2-phenylindole dihydrochloide (DAPI) (Santa Cruz, 1:1000). Secondary antibodies used in this study were bought from Jackson ImmunoResearch, and then were diluted at 1:500. For LMB (Sigma) treatment, S2 cells were treated with LMB at a final concentration of 5 nM for 2 h before cells were harvested for mRNA-cap localization assay. For *in situ* hybridization assay, the primers for *mRNA-cap* sub-cloning as follows: *mRNA-cap* upstream 5′-GTTATATGATGCTCATCGAT-3′; *mRNA-cap* downstream 5′-TTAATTTCCTAATGTCGGGT-3′; the corresponding of cDNA of *mRNA-cap* was cloned into pBluescript vector. *mRNA-cap* probe was prepared according to the instruction of the DIG RNA labeling kits (Roche).

### Cell culture, Transfection, RNAi interference, Immunoprecipitation, and Western blot assay

S2 cells were maintained at 25 °C in Schneider′s *Drosophila* Medium (S9895, Sigma) supplemented with 10% FBS (F0718, Gibco) and 1% penicillin/streptomycin (P0781, Sigma). Transfection was performed using calcium phosphate according to the manufacturer’s instructions (Invitrogen). Usually S2 cells are transfected in 10-cm plates with no more than 20 μg of total DNA for an *ubiquitin-Gal4* construct and other co-transfected pUAST expression vectors. 48 hrs after transfection, cells are harvested for immunoprecipitation and western blot analysis with standard protocols as previously described^40^. NIH/3T3 cells were cultured at 37 °C in an atmosphere of 5% CO_2_, in Dulbecco’s modified Eagle’s medium (DMEM; Life Technologies) supplemented with 10% FBS (F0718, Gibco) and 1% penicillin/streptomycin (P0781, Sigma). Indicated plasmids were transfected into cells with Lipofectamine 2000 (Invitrogen) according to manufacturer’s instructions. The following antibodies were used for immunoprecipitation and immunoblotting: mouse anti-Myc (9E10) (Santa Cruz, 1:5000), mouse anti-HA (Santa Cruz, 1:5000), mouse anti-Flag (M2) (Sigma, 1:5000), mouse anti-β-actin (GenScript, 1:10000), rabbit anti-PKAα (Santa Cruz, 1:500), rabbit anti-PKAIβ (Santa Cruz, 1:500), goat anti-rabbit HRP (1:10000; Jackson ImmunoResearch) and goat anti-mouse HRP (1:10000; Jackson ImmunoResearch). For RNA interference experiment, dsRNA was generated through *in vitro* transcription by using the MEGAscript T7 kit (Ambion). S2 cells were cultured in serum-free medium containing dsRNA for 12 hrs at 25 °C before transfection with DNA constructs. Then the culture medium was changed to serum medium and additional culturing for 48 hrs^63^. The primer sequences of mRNA-cap dsRNA are as follows: upstream 5′-GTTATATGATGCTCATCGAT-3′, downstream 5′-TTAATTTCCTAATGTCGGGT-3′. Western blot was performed as the standard protocols (described in Molecular Cloning). To silence RNGTT expression in 3T3 cells, the RNGTT siRNA was used (RiboBio). The sequence was 5′-GGAACCAUUUAGCGUCAGAdTdT-3′. All siRNA duplexes were transfected at a final concentration of 100 nM and maintained in serum-free medium for 4–6 hrs at 37 °C before transfection with indicated DNA constructs.

### Real-time quantitative PCR (RT-qPCR)

For RT-qPCR, RNA of wing discs was isolated by using Trizol reagent (Invitrogen) and then reverse-transcribed by Prime-Script RT reagent kit (TaKaRa). Finally, the real-time PCR was done using the SYBR Premix Ex Taq (TaKaRa) according to the instrument of StepOnePlus (Applied Biosystem). Standard RT-qPCR primers for *Drosophila mRNA-cap* (upstream: 5′-AAAAACCATTTAAAGCGC-3′ and downstream 5′-GCTTTGCGCGATCACTAGC-3′), *ci* (upstream: 5′-CCTCTTGCGTATTCTGAATT-3′ and downstream: 5′-GAATCTGATGTTCCACCCGT-3′), *smo* (upstream: 5′-CAGCTATACAGCCCTTTTTG-3′ and downstream: 5′-CACTGGCCAGTTCCGTTGAA-3′), *PKA-C1* (upstream: 5′-CACGAAAGACTATTATGCC-3′ and downstream: 5′-CGCGTAGAAGCGCGAGTGCGGCTCCG-3′), *PKA-C3* (upstream: 5′-GCCGCTCGAACGGCCGAAG-3′ and downstream: 5′-GGTGGTGGTGGTGGTGGCGG-3′), *PKA-R1* (upstream: 5′-GCGTTCTGAGCAGGGCGAGG-3′ and downstream: 5′-CCCAACACGCGTTCAAATC-3′), *PKA-R2* (upstream: 5′-ATATGCCCAGAGCGGCCACCGTGC-3′ and downstream: 5′-GGCGGC GTCACCCTGTTTGATG-3′) and *actin* (upstream: 5′-GTACCCCATTGAGCACGGTA-3′ and downstream: 5′-CGAACATGATCTGGGCATC-3′) were synthesized by GenScript. *actin* was used as a control.

### Luciferase reporter assay

In mammalian cells, Hh reporter assay was performed as previously described^64^. 48 hrs after transfection, cells were harvested and washed once with PBS, lysed in passive lysis buffer and luciferase activity was measured using a Dual Luciferase Assay Kit (Promega) as per manufacturer′s instructions. All luciferase activity data are presented as means ± SD of values from at least three experiments.

### Protein kinase and cAMP Assay

For measuring PKA activity, about 400 wing discs were used as a sample, which were dissected in the 1.5 ml tube with PBS on ice and kept at 4 °C. After centrifugation at 2,000 × g for 3–5 min at 4 °C, the PBS was removed, instead 500ul cold sample preparation buffer was added. Then the discs were sonicated for 30–60 seconds or 15 seconds x 3 times. The samples were kept cool and cell suspensions were avoided foaming during the sonication. Next, cytosol fraction was separated by centrifugation at 100,000 × g for 1 hour at 4 °C. Finally, the supernatant was used to measure the PKA activities according to the protocol of MESACUP Protein Kinase Assay kit (MBL, No.5230). The cAMP amount was measured by cAMP Direct Immunoassay Kit (Biovision, No.K371-100) with about 400 wing discs, which were collected as described above and frozen quickly with liquid nitrogen. Then 200ul 0.1 M HCl was added, the sample was blended with homogenizer on ice. After centrifugation at 100,000 × g for 5 min, the supernatant was used to test the cAMP amount. The PKA activity and cAMP level were measured from *MS1096* control wing discs or wing discs expressing *mRNA-cap* RNAi with *MS1096* driver.

### Statistical analysis

Imaging data were analyzed using Image J. Statistical tests were performed in GraphPad Prism 5. Data presented are the average of three independent experiments and error bars represent SD. A value of P < 0.05 is considered statistically significant by using Student′s t test.

## Electronic supplementary material

Supplementary information
