# Supplementary material for: Capping Enzyme mRNA-cap/RNGTT Regulates Hedgehog Pathway Activity by Antagonizing Protein Kinase A
Source: Sci Rep. 2017 Jun 6;7:2891. doi: 10.1038/s41598-017-03165-2 (PMC5460166; doi:10.1038/s41598-017-03165-2)

Supplementary Information

Capping Enzyme mRNA-cap/RNGTT Regulates Hedgehog Pathway Activity by Antagonizing Protein Kinase A

Ping Chen[1,#], Zizhang Zhou[1,#], Xia Yao[1], Shu Pang[1], Meijing Liu[1], Weirong Jiang[1], Jin Jiang[2,3*] and Qing Zhang[1,*]

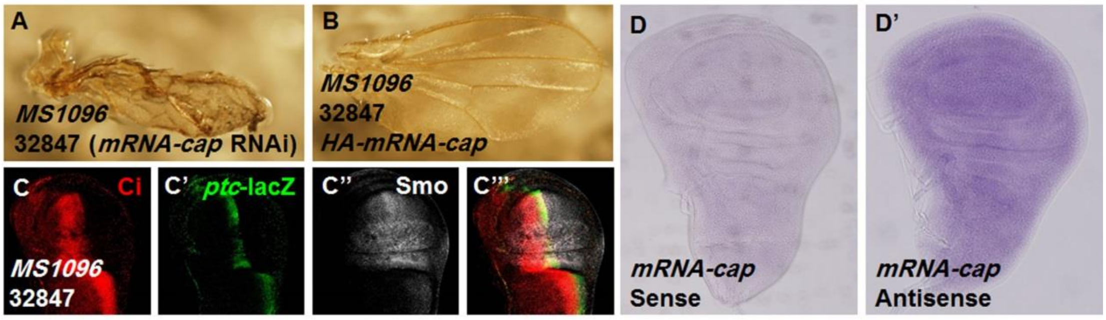

Figure S1. Adult wing morphology and Hh pathway activity are affected by *mRNA-cap* RNAi. (A) Knockdown of *mRNA-cap* by *mRNA-cap* RNAi line 32847 caused abnormal wing phenotype. (B) *HA-mRNA-cap* expression rescued the wing phenotype induced by the *mRNA-cap* RNAi line 32847. (C-C''') Expression of 32847 with *MS1096* downregulated Ci and *ptc*-lacZ levels but upregulated Smo levels. (D-D') *mRNA-cap* expression pattern of wing discs was shown by *in situ* hybridization assay. Sense probe was used as control. All images are representative of three independent experiments.

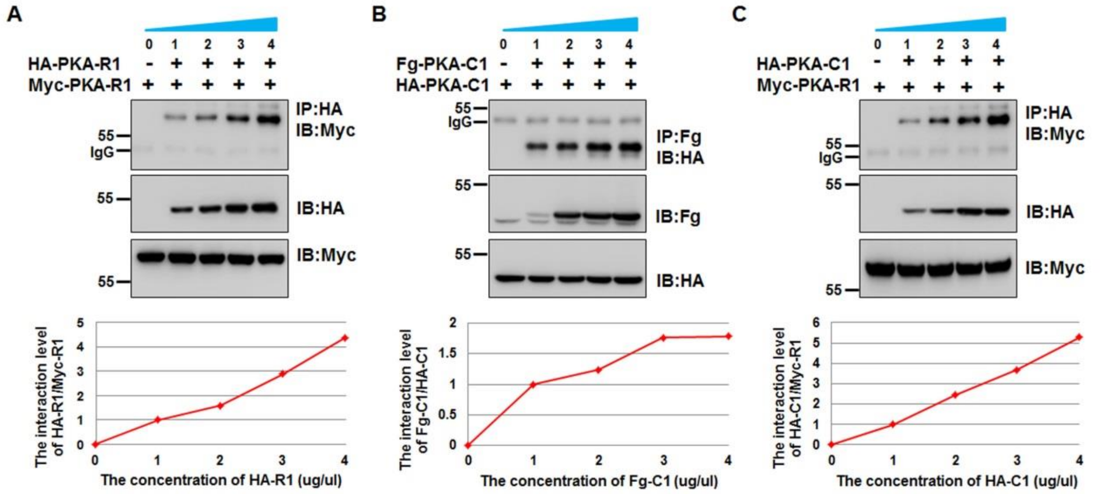

Figure S2. The input titrations assay. (A-C) HA-PKA-R1 (A), Fg-PKA-C1 (B) and HA-PKA-C1 (C) were expressed at different concentrations (0, 1, 2, 3, 4 ug) while Myc-PKA-R1 (A), HA-PKA-C1 (B) and Myc-PKA-R1 (C) were expressed at a fixed (4ug) concentration. To keep the interaction of each specific pair is in the linear ranges, we chose the middle amount of 2ug

plasmids to do the experiments of Fig. 5J-M. All images are representative of three independent experiments.

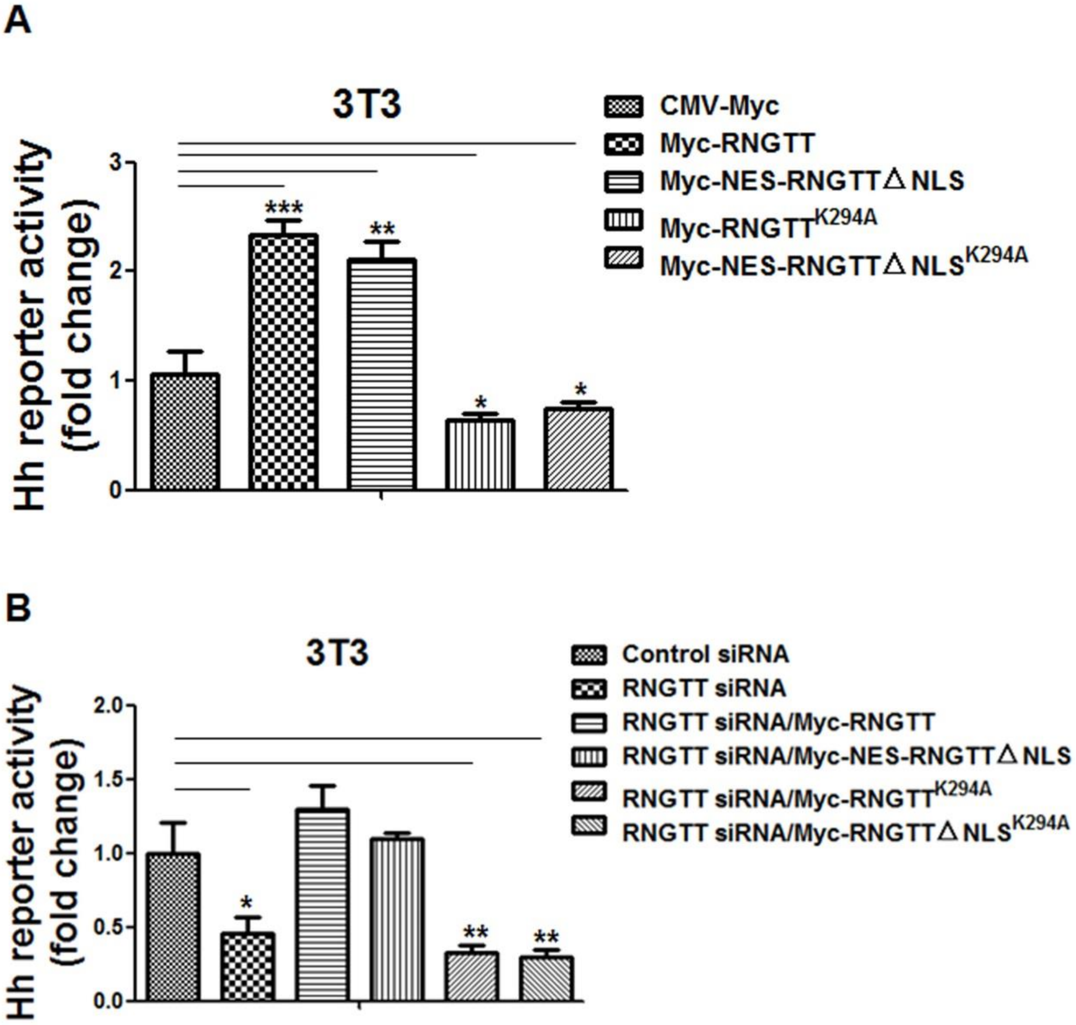

Figure S3. The cytoplasmic localized RNGTT regulates the Hh pathway. (A) In the 3T3 cells, both the wild type RNGTT and cytoplasmic localized RNGTT (*Myc-NES-RNGTT⊿NLS*) upregulated the Hh activities, while the mutants of RNGTT^{K294A} and cytoplasmic localized RNGTT^{K294A} (*Myc-NES-RNGTT⊿NLS^{K294A}*) downregulated the Hh activities. (B) In *RNGTT* knockdown 3T3 cells, expression of the wild type RNGTT and cytoplasmic localized RNGTT but not their mutant forms rescued Hh pathway activity. Data presented are the average of three independent experiments and error bars represent SD. $^{*}P < 0.05$, $^{**}P < 0.01$, $^{***}P < 0.001$, t-test.

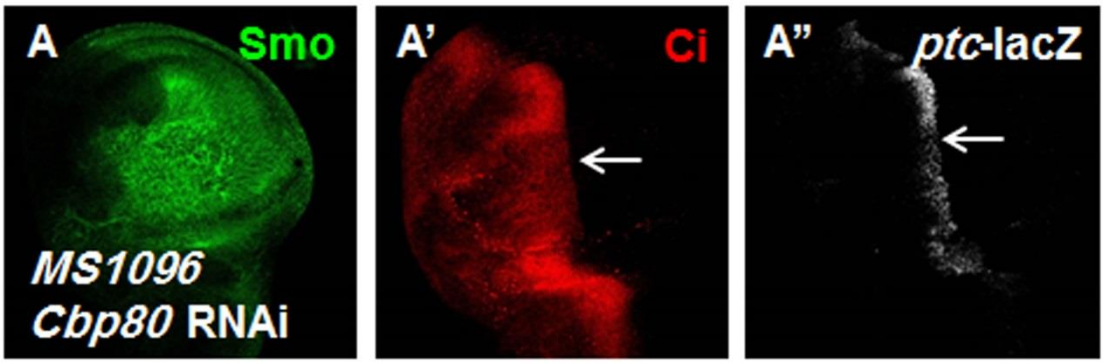

Figure S4. Knockdown of *Cbp80* decreases the Hh signaling activity. (A-A") Wing discs expressing *Cbp80* RNAi with *MS1096* were immunostained for Smo (green), Ci and *ptc*-lacZ. Knockdown of *Cbp80* with *MS1096* elevated Smo but attenuated the expression of Ci and *ptc*-lacZ. Arrows indicate the decrease of Ci and *ptc*-lacZ. All images are representative of three independent experiments.

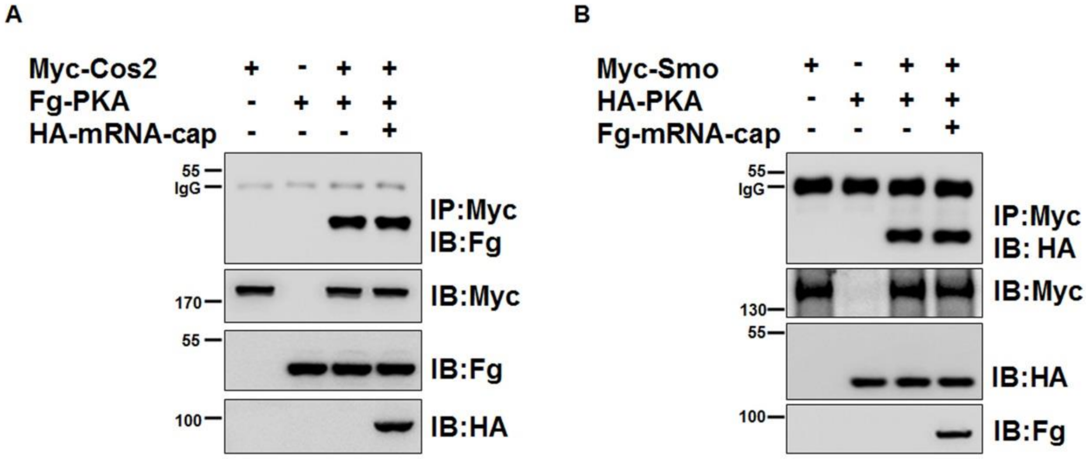

Figure S5. mRNA-cap doesn't influence the interaction of PKA-Cos2 and PKA-Smo. (A-B) mRNA-cap did not antagonize the association between PKA and Cos2 or Smo in S2 cells. All images are representative of three independent experiments.

Figure. 5H

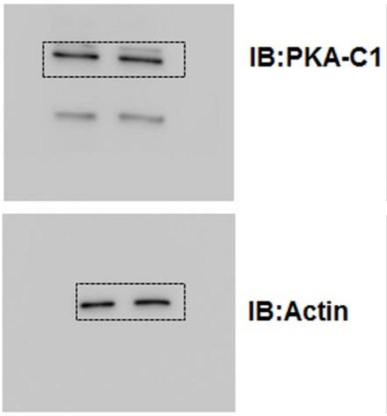

Figure. 5I

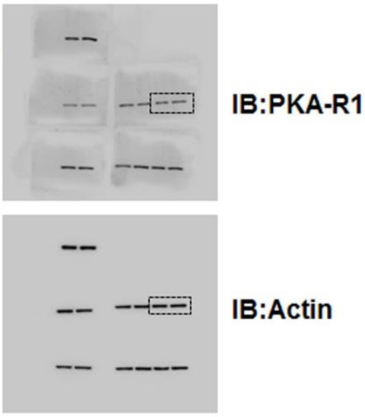

Figure. 5J

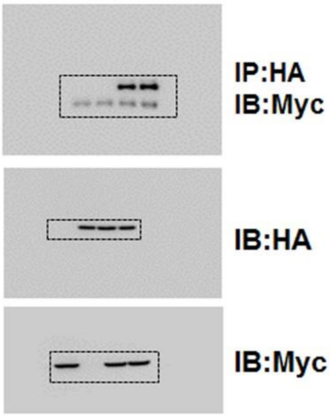

Figure. 5K

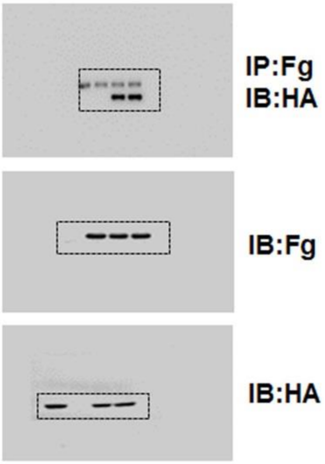

Figure. 5L

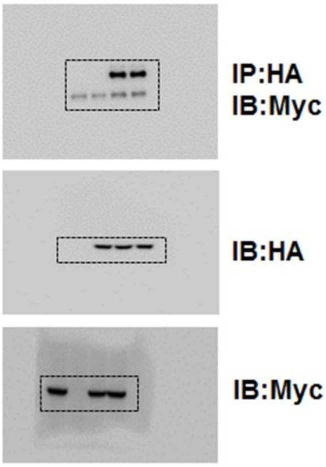

Figure. 5M

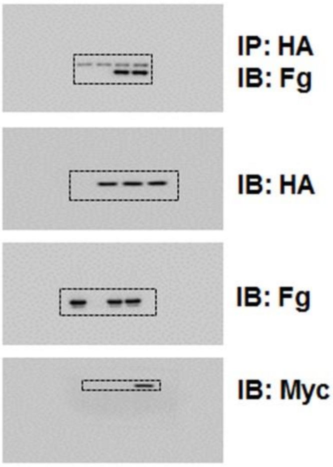

Figure-S2A

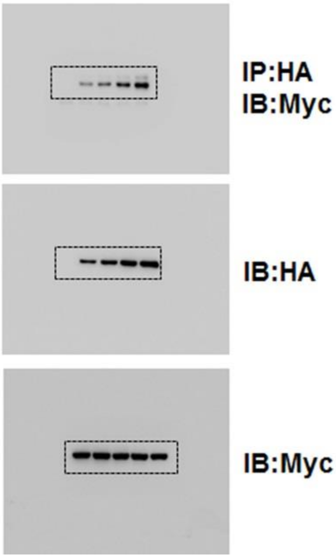

Figure-S2B

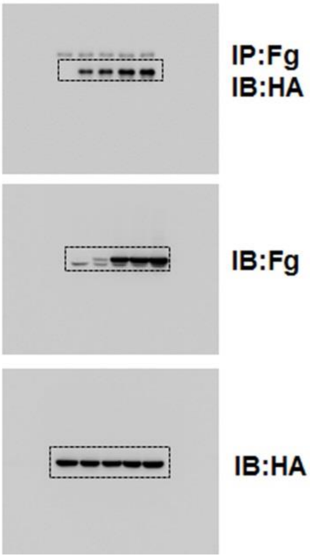

Figure-S2C

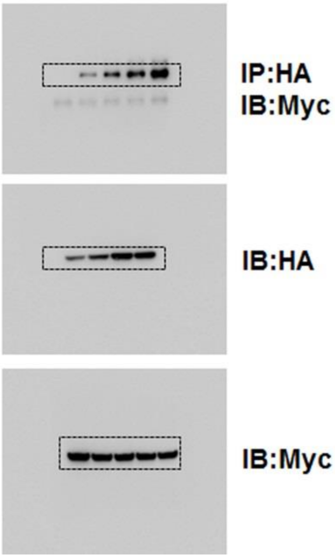

Figure-S5A

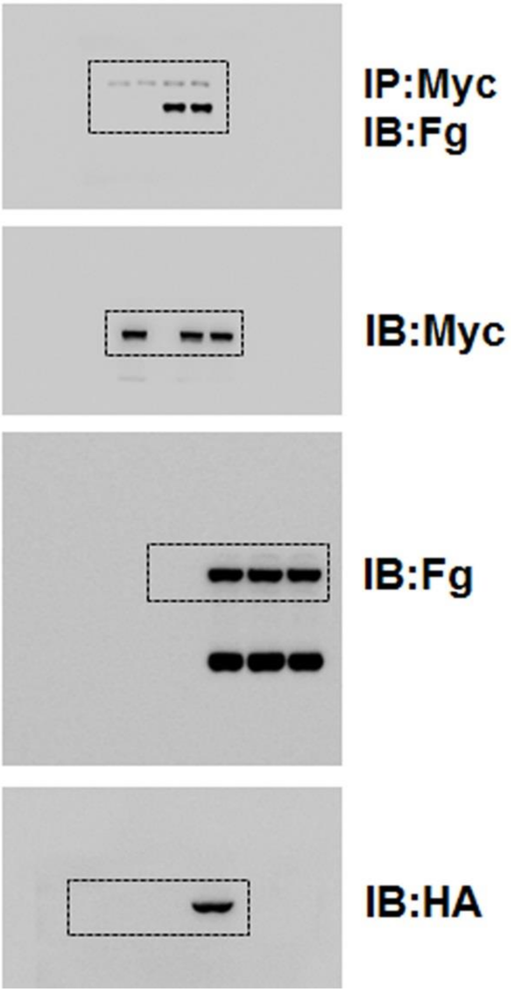

Figure-S5B

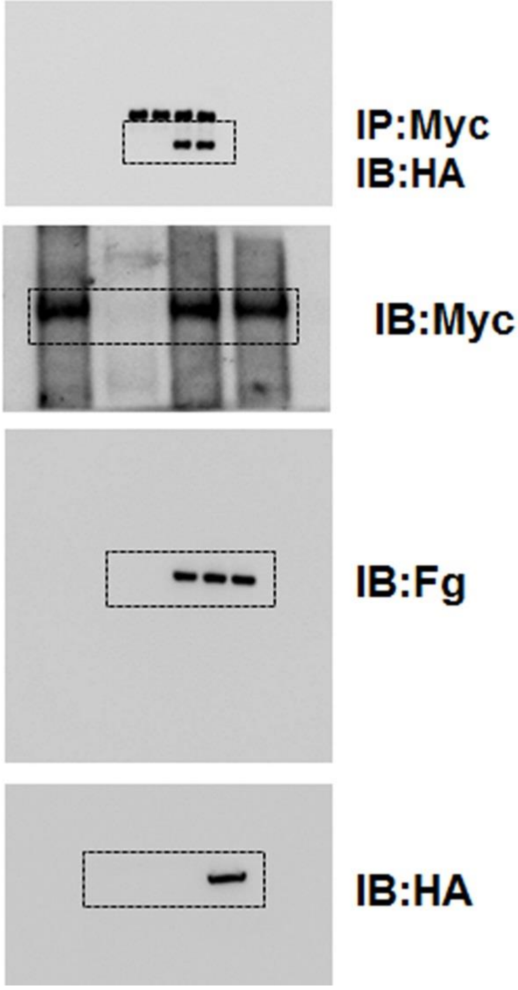

Supplement: Supplementary file 1 — Supplementary information [file 41598_2017_3165_MOESM1_ESM.pdf]
